# Convulsions in Children

**Published:** 1857-08

**Authors:** J. J. Scott

**Affiliations:** Oxford, Mississippi


*Convulsions in Children.* By J. J. Scott, M. D., of Oxford, Mississippi.

The very thought of convulsions or "fits," carries with it terror to the friends of those threatened with spasmodic disease. This is not so much to be wondered at, when we remember that the subjects of convulsions, not only are thrown into a state resembling articulo mortis itself, but that certain forms of spasmodic affections, are heart-rending to look upon, and hopeless as regards a cure. Many cases of Epilepsy, Tetanus and hydrophobia are of this kind. Instances of such being indelibly impressed upon the mind, it is not surprising that the greatest dread and alarm is excited, not only in the mother, but the physician also, where spasms ensue upon comparatively trivial causes, which may be readily removed, and the sufferer restored to consciousness, without leaving any particular liability to its return.

Worms, hooping cough, and in fact any inflammatory or painful disease, in children, may lead to muscular spasm, having, in many instances, all the characteristics of epilepsy.

In children, from the great mobility of the nervous system, any protracted or excessive irritation in the alimentary canal or elsewhere, is liable to induce that state of the nervous centres from which convulsions result. This may be brought about without any actual irritation or congestion of the centres themselves, but by means of the strong sympathy and intimate connection of all parts of the body, with the great source of nervous power.

Temporary disturbance of the brain itself, from a general febrile excitement, and by mechanical causes, producing congestion and compression of the organ, lead to these distressing symptoms, which, although it is possible for a permanent convulsive habit to renew, generally passes away with the cause of the disturbance.

The mechanical force applied to the large blood vessels situated in the thorax and abdomen in the act of coughing, made violent and protracted by hooping-cough, favors, in children of full habit, unpleasant congestion of the brain.

Inflammatory diseases of the throat and other parts contiguous to the brain, by the excitement and consequent flow of blood to neighboring parts, sometimes result in temporary congestion and depression of this organ.

Such cases sometimes excite great alarm, when nothing is required but to restore the equilibrium of the circulation.

A case in point: On the 28th April was called to see a little boy, aged 3 years, who had for several days been affected with croup, but to within a short time before I saw him, was supposed to be convalescent. I found the little patient violently convulsed. The spasmodic muscular contraction continued until, by general warm bath, cold applications to the head, cups to the temples, and sinapisms, general relaxation and an equalization of the circulation was produced.

Many similar cases are met with, in which convulsions are found to arise from such causes. Sometimes it is necessary to push our investigations and treatment still farther. When local inflammations, the irritations from teething, worms, &c., are suspected as the cause, prompt attention should, of course, always be given to such causes, lest, by allowing, from the continuance of the exciting cause, the repetition of the convulsion, the nervous system may become so impressed that the habitual recurrence of spasm may follow. There is, perhaps, no doubt that permanent spasmodic affections exist from these very causes, which might have been prevented by early attention to the sources of irritation, by which the habit was induced.

